# Supplementary material for: Analysis of biopsies of gastric cancer, intestinal and diffuse, and non-atrophic gastritis: an overview of loss of heterozygosity in Mexican patients
Source: PeerJ. 2025 Feb 25;13:e18928. doi: 10.7717/peerj.18928 (PMC11869887; doi:10.7717/peerj.18928)
Supplement: Supplemental Information 6 [file peerj-13-18928-s006.pdf]

MIAME Checklist

Part 1 Experiment description

- Tissue samples were obtained from cancer gastric and gastritis non-atrophic patients.
-
- Diffuse gastric cancer and intestinal gastric cancer diagnoses, and subjects with non-atrophic gastritis as controls.
-
- Diffuse gastric cancer (DGC, n = 7) and intestinal gastric cancer (IGC, n = 7) diagnoses, and subjects with non-atrophic gastritis (NAG, n = 7) as controls tissues used for slide.
-

Table 1 shows the general characteristics of the 21 patient samples, age (mean ± SD, 59.61 ± 15.94 years), sex (Female 23.8% and Male 76.2%), and the percentage of neoplastic cells for tumor tissues ranging between 50 and 70%. One IGC patient and three with NAG were positive for *H. pylori*. Data from the Tumor size, Number of nodes, and Metastasis (TNM) classification system are presented.

Part 2 Array design

The CytoScan® Array was designed by empirically selecting probes from a pool of over 20 million probes and then further screening them with over 3,000 samples to choose those that performed best for whole-genome cytogenetic applications.
The design is optimized for balanced whole-genome coverage, enabling high- resolution DNA copy number analysis with precise breakpoint accuracy as well as high-density SNP coverage for loss of heterozygosity (LOH)/absence of heterozygosity (AOH), long contiguous stretches of homozygosity (LCSH), and uniparental isodisomy (UPD) detection.
Affymetrix' proprietary manufacturing technology produces arrays that are highly reproducible between each batch with no risk of probe dropout inherent in some manufacturing techniques.
Covers all 36,000 RefSeq genes, including 12,000 OMIM®, all ICCG (ISCA) and Decipher constitutional regions, and Sanger cancer genes

- **Array series:** Affymetrix® CytoScan™ microarray (Affymetrix; Thermo Fisher Scientific, Inc.)
- **Deconvoluted spot list with gene names:** Whole-genome cytogenetic applications. Affymetrix® CytoScan™ microarray.
- **Array type:** Human gDNA
- **Array size:** Cytoscan HD Suite provides the broadest coverage and highest throughput for detecting chromosomal aberrations. CytoScan HD Suite has greater than 99% sensitivity and can reliably detect 25-50 kb copy number changes across the genome with high specificity with single nucleotide polymorphism (SNP) allelic corroboration. With over 2.6 million copy number markers, Cytoscan HD Suite covers all OMIM™ and RefSeq genes. The proprietary manufacturing technology produces highly reproducible batch-to-batch arrays with no risk of probe loss inherent to bead manufacturing techniques.
- **Slide type (and coating):** HD Array, GeneChip Probe Array.

<u>Part 3 Samples</u>

gDNA was restriction digested, PCR amplified, fragmented, labeled and hybridized to each array according to the manufacturer's instructions. *CytoScan^{TM} Assay Manual Workflow User Guide*.

- **Cy3/Cy5 labels for tissues:** Biotin/Streptavidin.

Dye swap? Or reference control?: Positive and negative controls.

- **Labelling protocol used:** As per manufacturer. *CytoScan^{TM} Assay Manual Workflow User Guide*.
-

Sample extraction protocol used: DNA extraction was done with a QIAamp® micro kit (QIAGEN) according to the manufacturer's instructions. The extractions were modified to include an initial incubation at 95°C for 15 min followed by 5 min at room temperature as described previously, before being digested with proteinase K for 3 days at 56°C in a water-bath with addition of fresh enzyme at 24 h intervals. *CytoScan^{TM} Assay Manual Workflow User Guide*.

Amount of sample labelled 250 ng gDNA

-

Part 4 Hybridizations

gDNA was restriction digested, PCR amplified, fragmented, labeled and hybridized to each array according to the manufacturer's instructions. *CytoScan^{TM} Assay Manual Workflow User Guide*.

- **Hybridization protocol:** hybridized to each array according to the manufacturer's instructions. *CytoScan^{TM} Assay Manual Workflow User Guide*.
- **ALL modifications and deviations from the protocol:** None
- **Manual hybridization or automatic chamber?** As per manufacturer. *CytoScan^{TM} Assay Manual Workflow User Guide*.
- **Number of slides done at the same time:** 2 or 4
- **Hyb time:** 16-18 h
- **Number of washes:** As per manufacturer. Affymetrix GeneChip® Command Console (AGCC, Version 3.2.2 or higher) to operate the fluidics station (GeneChip® Fluidics Station 450) *Assay Manual Workflow User Guide*.
- **Amount of labelled sample hybridized:** As per manufacturer. *CytoScan^{TM} Assay Manual Workflow User Guide*.
- **Labelling efficiency:** As per manufacturer. *CytoScan^{TM} Assay Manual Workflow User Guide*.

Part 5 Measurements

Which version of scanner software used: GeneChip® Scanner 3000 7G The instrument control application: Affymetrix GeneChip® Affymetrix — Command Console v.3.2.2 or higher

- **Instrument model numbers:** GeneChip® Scanner 3000 7G

- The archives were saved on .CEL and analyzed on Chromosome Analysis Suite (ChAS) 4.3.0.71 for each sample (GEO: GSE117093 and BioProjet: PRJNA481039)

  The construction of the GRCh38 genome (December 2013) was used as a reference model and CytoScanHD_Array.na36.annot.db file for annotation. Data processing was based on the segmentation algorithm, where the Log2 ratio for each marker was calculated relative to the reference signal profile. To calculate the LOH, the data were normalized to baseline reference intensities using ChAS reference model including 284 HapMap samples and 96 healthy individuals. The Hidden Markov Model (HMM) was used to determine the LOH segment calls. The customized conditions were filtered to determine LOH, 3 Mb, and 50 Single Nucleotide Polymorphisms (SNPs). The Median Absolute Pairwise Difference (MAPD) and the Single Nucleotide Polymorphism Quality Control (SNPQC) score were used as the quality control parameters. Only samples with values of MAPD < 0.25 and SNPQC > 15 were included in the further analysis.

  Part 6 Normalization controls

- **Hypothesis:** We will find differences in the loss of heterozygosity in samples of DGC, IGC, and NAG aggregators, that is, between gastric cancer (diffuse and intestinal) and non-cancer. This will help us generate knowledge of the biology of cancer to predict its early diagnosis, progression, and response to treatment.
- **Gene expression patterns found:** Described in the paper, results and discussion.
- **Controls used, normalization methods used (see above):** Described in the paper, material and methods and As per manufacturer. *CytoScan^{TM} Assay Manual Workflow User Guide*.
-

Taken from the Miame paper found here: http://www.nature.com/cgi-taf/DynaPage.taf?file=/ng/journal/v29/n4/full/ng1201-365.html
