# Supplementary material for: Analysis of biopsies of gastric cancer, intestinal and diffuse, and non-atrophic gastritis: an overview of loss of heterozygosity in Mexican patients
Source: PeerJ. 2025 Feb 25;13:e18928. doi: 10.7717/peerj.18928 (PMC11869887; doi:10.7717/peerj.18928)
Supplement: Supplemental Information 7 [file peerj-13-18928-s007.pdf]

STROBE Statement—Checklist of items that should be included in reports of ***case-control studies***

	Item No	Recommendation
**Title and abstract**	1	(*a*) Analysis of biopsies of gastric cancer, intestinal and diffuse, and non-atrophic gastritis: an overview of loss of heterozygosity in Mexican patients
		(*b*) Worldwide, gastric cancer (GC) is a common malignancy with the highest mortality rate among digestive system diseases. The present study of GC and loss of heterozygosity (LOH) is relevant to understanding tumor biology and establishing essential aspects of cancer. Here, DNA samples from Mexican patients with diffuse GC (DGC), intestinal GC (IGC), or non-atrophic gastritis (NAG; control) were purified, and whole-genome high-density arrays were performed. Posteriorly, LOH was identified among the tissue samples, and cancer genes and signaling pathways were analyzed to determine the most altered. Detailed bioinformatics analysis was developed to associate LOH with the Hallmarks of Cancer according to their frequency in patient samples, participation in metabolic pathways, network interactions, and enrichment of Cancer Hallmark genes. LOH-genes in GC were PTPR, NDUFS3, PAK3, IRAK1, IKBKG, TKTL1, PRPS1, GNAI2, RHOA, MAPKA, and MST1R. Genes that stand out at NAG involve proliferation and growth; those at IGC trigger genomic instability, tissue invasion, metastasis, and arrest of cell death; and those at DGC involve energy metabolism, the destruction of immune evasion, and replicative immortality. Other events, such as sustained angiogenesis, were similar between NAG-IGC-DGC. Together, these are molecular, cellular, and metabolic events that must be monitored in GC patients. Our findings must be validated to develop molecular tests for diagnosis, prognosis, treatment response, and, most importantly, screening tests.
**Introduction**		
Background/rationale	2	Gastric cancer (GC) ranks fifth in the world according to incidence and mortality (1). GC is a multifactorial disease with environmental and genetic factors impacting its occurrence and development. The GC incidence rate rises progressively with age; the average age for diagnosis is 70 years, a small portion of gastric carcinomas (10%) are detected at younger ages (45 or less) and becomes good chances to look for GC early genetic alterations or carcinogenesis pathways since those patients are less exposed to environmental facts. Carcinogenesis is a multistage process with a progressive development that involves gene mutations and epigenetic alterations (2).<br>GC molecular classification involves four subtypes: tumors with chromosomal instability (CIN), tumors with microsatellite instability (MSI), genomically stable tumors (GS), and EBV-positive tumors (3). CIN is one of the significant genomic instability pathways involved in gastric carcinogenesis, and it is characterized by losses or gains of whole chromosomes that result in aneuploidy. CIN may also involve changes in portions of chromosomes, which include allelic losses like loss of heterozygosity (LOH), gene deletions, amplifications (4, 5) or rearrangement (6).<br>Also, two main GC histotypes are recognized: intestinal and diffuse. Although most of the described genetic alterations have been observed in both types, different genetic pathways have been hypothesized. Genetic and epigenetic events, including LOH, have mostly been reported in intestinal-type gastric carcinoma (IGC) and its precursor lesions, whereas LOH mutation (p53) are implicated in the diffuse-type gastric cancer (DGC) (7).<br>LOH has been identified as an etiological factor in CIN, which is a Hallmark of

		Cancer, including GC (6). LOH involves the loss of one of the two gene alleles in a cell, which can lead to the inactivation of tumor suppressor genes and contribute to the development and progression of cancer (7). There are two types of LOH: (1) copy number loss LOH (CNL-LOH), which implies the loss of alleles, for tumor suppressor gene as an example, and (2) copy number neutral LOH (CNN-LOH), without any affecting function nor contributing to the disease development (8). A complete or partial deletion of a chromosome leads to CNL-LOH, while CNN-LOH is mainly caused by acquired uniparental disomy (UPD) and genetic conversion and occurs without a net change in copy number (9) (10).<br>Various molecular techniques have been used to investigate the role of LOH in GC (11), such as polymerase chain reaction (PCR), microsatellite marker sites PCR (12), multiplex ligation-dependent probe amplification (MLPA) (13), polyacrylamide gel electrophoresis (PAGE) (14), silver stain (15), exome sequencing (16), Illumina (17) and Affymetrix (18) microarrays. The identification of LOH-events can be assessed by gene expression using RNA-Seq and RT-PCR (19) and protein expression with immunohistochemistry (IHC) (18). LOH has also been correlated with CpG hypermethylation processes in patients with GC (20). The LOH-genes associated with CG most frequently reported are TP53 (21), PTEN (16), RB1, and BRCA1 (22). The loci involved in tumor suppression are located on chromosomes 1, 3, 7, 8, 11, 12, 13, 18, and 22 (7, 17, 23). Different scores have also been proposed to establish risk or diagnosis based on LOH (22). For the above, our interest arose in determining the LOH patterns in a group of IGC, DGC, and non-atrophic gastritis (NAG) samples to find guidelines for therapeutic targets and data that enrich the knowledge of cancer biology.
Objectives	3	Our interest arose in determining the LOH patterns in a group of IGC, DGC, and non-atrophic gastritis (NAG) samples to find guidelines for therapeutic targets and data that enrich the knowledge of cancer biology.
**Methods**		
Study design	4	Samples. Tissue samples were obtained from 21 patients (5 females and 16 males) that met the criteria for diffuse gastric cancer (DGC, n = 7) and intestinal gastric cancer (IGC, n = 7) diagnoses, and subjects with non-atrophic gastritis (NAG, n = 7) as controls. To guide the investigation of relevant alterations, the present analysis focused on LOH-events present in at least three patients (cut-off, ≥ 3 patients; ≥ 40% samples) to identify the most relevant GC alterations (Table 1)
Setting	5	The samples come from a repository
Participants	6	(*a*) Describe on Table 1 of the manuscript
		(*b*) Not applicable
Variables	7	Not applicable
Data sources/ measurement	8*	*LOH processing.* The raw intensity files (.CEL) retrieved from the commercial platform Affymetrix® CytoScan™ microarray (Affymetrix; Thermo Fisher Scientific, Inc.), were analyzed using Chromosome Analysis Suite (ChAS) v4.3.0.71. The construction of the GRCh38 genome (December 2013) was used as a reference model and CytoScanHD_Array.na36.annot.db file for annotation. Data processing was based on the segmentation algorithm, where the Log2 ratio for each marker was calculated relative to the reference signal profile. To calculate the LOH, the data were normalized to baseline reference intensities using ChAS reference model including 284 HapMap samples and 96 healthy individuals. The Hidden Markov Model (HMM) was used to determine the LOH segment calls. The customized conditions were filtered to

		determine LOH, 3 Mb, and 50 Single Nucleotide Polymorphisms (SNPs). The Median Absolute Pairwise Difference (MAPD) and the Single Nucleotide Polymorphism Quality Control (SNPQC) score were used as the quality control parameters. Only samples with values of MAPD < 0.25 and SNPQC > 15 were included in the further analysis.<br>*Bioinformatics analysis.* To generate a list of genes and frequencies for altered regions, Practical Extraction and Report Language (Perl) scripts (25) were developed to load the LOH segment data files generated by ChAS 4.3.0.71 for each sample, including chromosomes and cytogenetic bands and Online Mendelian Inheritance in Man information, and haploinsufficiency predictions version 3 information from the DatabasE of Genomic Variation and Phenotype in Humans, using Ensemble Resources (DECIPHER v11.25). Custom scripts were developed in Perl v5.32 to obtain the frequency of LOH -genes and -cytobands and the length of events.<br>The genes altered in at least three patients (cut-off, ≥ 3) with DGC, IGC, and NAG were included for analysis and visualization. The genes were compared by generating Venn diagrams with the Jvenn server (26). Cancer Hallmarks enrichment analysis (p.adjust < 0.05) was performed with a collection of 6,763 genes (https://cancerhallmarks.com/). The results were reviewed using the Catalog of Somatic Mutations in Cancer database (COSMIC v100) (27), the Hallmarks of Cancer database (HOCdb database) (28).<br>Reactome v88 performed a metabolic pathway enrichment analysis (29), considering those results significant with values less than 0.05 in the false discovery rate (FDR). Finally, an interaction network was generated based on metabolic pathways and genetics, as well as physical and functional associations to establish the Cancer Hallmarks associated with the profile of LOH-genes IGC, NAG, and "core" IGC-DGC-NAG. Furthermore, these were determined using the STRING v12.0 prediction server (30) and Cytoscape v.3.10.0 (31), including manual annotation of their corresponding the Cancer of Hallmarks (adhesion, angiogenesis, inflammation, migration, metastasis, morphogenesis, proliferation, and survival)
Bias	9	Not applicable
Study size	10	Based on the inclusion, exclusion and elimination criteria
Quantitative variables	11	Explain on point 8
Statistical methods	12	(*a*) Explain on point 8
		(*b*) Explain on point 8
		(*c*) Explain on point 8
		(*d*) Explain on point 8
		(*e*) Explain on point 8
**Results**		
Participants	13*	(a) Explain on point 4
		(b) Not applicable
		(c) Not applicable
Descriptive data	14*	(a) Material and methods section of the mansucript
		(b) Not applicable
Outcome data	15*	Tables 1and 2 of the manuscript and supplementary tables 1 and 3
Main results	16	(*a*) Sample characteristics. This study included tissue samples from 21 Mexican patients without treatment (naïve). Patient samples included seven DGC cases, seven for IGC, and seven more corresponding to NAG (as controls). The .CEL files and their

raw intensity values obtained from the microarrays were deposited in the Center for Biotechnology Information (NCBI), with the accession key GSE117093 and BioProjet PRJNA481039.

Table 1 shows the general characteristics of the 21 patient samples, age (mean ± SD, 59.61 ± 15.94 years), sex (Female 23.8% and Male 76.2%), and the percentage of neoplastic cells for tumor tissues ranging between 50 and 70%. One IGC patient and three with NAG were positive for H. pylori. Data from the Tumor size, Number of nodes, and Metastasis (TNM) classification system are presented.

Genomic detection of LOH. The LOH of the patients was estimated using the analysis described before, a meticulous process based on regions where the preponderance of SNPs does not display heterozygosity. Table 2 shows a summary of the chromosomes with the highest involvement frequency concerning the number of events (coincidences) at the LOH-regions but not strictly perfect in the chromosomal coordinates.

Our data, which includes the megabase pairs cumulative length (Mbp-cl) of our tissue samples, were also reviewed (Table S1), the LOH-gene frequency data, chromosomes, and cytobands, is presented. Table S2 displays the accumulated LOH-length (Mb) values per chromosome, to determine if more extended losses indicate more damage.

In DGC patients, the affected chromosomes with Mbp-cl and the specified number of LOH-events were 6, 8, 16, and X; at IGC, they were chromosomes 3, also 16, and 17. Chromosomes 6 and 8 are associated with DGC, while 3 and 17 are associated with IGC (Table 2 and S2). Following, we found that there are 3,361 LOH-genes in DGC (Table S1); chromosomes Xq11.1/Xp22.23 in 7/7 male patients and chromosome 16p11.2 in 6/7 male patients (Tables 3 and S3) were the most altered.

In IGC, 2,490 LOH-genes were determined (Table S1) with chromosomes Xq11.1 for 7/7 patients, Xp22.33 for 6/7 patients, 16p11.2 for 6/7 patients, 3p21.31 for 5/7 patients and 17q22 for 3/7 patients (Table 3 and Table S3) as the most altered.

Finally, in NAG 4,748 LOH-genes were determined (Table S1). Chromosomes Xq11.1 for 7/7 patients, 16p11.2 for 7/7 patients, and Xp22.33 for 4/7 patients (Table 3 and Table S3) were the most altered.

Interestingly, LOH lengths do not look relevant for carcinogenesis, and 5-10 Kbp LOH-lengths were more common and frequent in DGC, IGC, and NAG (Table S4).

To identify the most relevant LOH in GC and NAG, we analyzed alterations occurring in at least three patients (cut-off ≥ 3). We found a similar pattern for total LOH, with more events in DGC (1157), IGC (1361), and NAG (1184). In addition, DGC had the highest number of genes affected in all samples, 7/7 (1132), followed by IGC (261) and NAG (34) (Table S1).

Gastric cancer genes associated with LOH. A Venn diagram was constructed to examine the LOH-GC-relevant genes of at least three patients (cut-off ≥ 3) of the DGC, IGC, and NAG. We determined 1153 shared LOH-genes between DGC-IGC-NAG. IGC had 207 unique affected genes, while NAG only showed 28 (Figure 1A and Table S5). From each subset (Figure 1A), those genes with matches according to the Cancer Hallmarks Genes database, a comprehensive resource that includes 6,763 genes, are shown 241 LOH-genes were found in DGC-IGC-NAG; IGC had 55

		affected unique genes and 13 genes in NAG. Figures 1B-D represent the enrichment of the Hallmarks of Cancer. The 241 common genes DGC-IGC-NAG present a greater number of Hallmarks than NAG, which showed fewer.<br><br>Functional pathway analysis. Using the LOH-genes-Hallmarks identified in each subset, metabolic pathways were predicted in Reactome using Homo sapiens as a model organism; in Table 4, those significant metabolic pathways (p-value < 0.05) and a brief general description are reported.<br><br>Correlation genes network. Cancer LOH-genes-Hallmarks associated with metabolic pathways were used to construct interaction networks (String, Figure 2). The connecting lines indicate associations by metabolic pathways, expression, localization, inferred interaction, genetic interactions, data mining, and neighborhood. Likewise, each node can be related to flags (events) related to report the Hallmarks of Cancer. In this way, the network formed among IGC-DGC-NAG had 31 nodes; the genes with the highest number of Hallmarks were PAK3, IRAK1, and IKBKG, and the genes with the highest number of connections were OPHN1, WAS, TKTL and PRPS1. In the ICG network, there were 29 nodes. The genes associated with Cancer Hallmarks were GNAI2, RHOA, MAPKAPK3, HYAL1, and CISH, while the most connected were RHOA, MST1R, and ATRIP. Finally, the network corresponding to NAG had five nodes, PTPRJ had the most significant number of Hallmarks, and the most connected was NUP160 (Table 5).
		(*b*) Not applicable
		(*c*) Not applicable

Other analyses	17	Not applicable
**Discussion**		
Key results	18	Here, 11 LOH-genes were determined, which were selected according to their apparition frequency in the analyzed samples, their participation in metabolic pathways (p-value < 0.05), their established interactions (networks), and their enrichment in Cancer Hallmarks genes database (p.adjust < 0.05). Thus, PTPRJ and NUP160 were determined into NAG samples, RHOA, GNAI2, and MAPKAPK3 for ICG, and no unique or relevant genes were identified for DCG. NAG LOH-genes that are relevant to carcinogenesis participate in proliferation and growth, while those for IGC are on genomic instability, tissue invasion, metastasis, and the arrest of cell death; and DGC genes are for energy metabolism, destruction of immune evasion, and replicative immortality. Other genes were shared between IGC and NAG-IGC-DGC, whose p values are close and could be considered similar LOH-events since they are involved in sustained angiogenesis. On the other hand, IGC genes also promote inflammation, and although the p-values are not significant, there was a difference in the NAG-IGC-DGC group. Then, those molecular, cellular, and metabolic LOH-alterations should be monitored in GC patients. These findings must be validated to develop tests with molecular profiles for diagnosis, prognosis, and response to treatment, as well as, most importantly, screening tests.
Limitations	19	Unbiased
Interpretation	20	They are explained in the article discussion
Generalisability	21	They are discussed in the article discussion
**Other information**		
Funding	22	The present study was supported by the Fondo de Investigación en Salud-Instituto Mexicano del

	Seguro Social (grant nos. FIS/IMSS/PROT/G16/1573 and FIS/IMSS/PROT/PRIO/13/027)., the financing was institutional with the original purposes.

*Give information separately for cases and controls.

Note: An Explanation and Elaboration article discusses each checklist item and gives methodological background and published examples of transparent reporting. The STROBE checklist is best used in conjunction with this article (freely available on the Web sites of PLoS Medicine at http://www.plosmedicine.org/, Annals of Internal Medicine at http://www.annals.org/, and Epidemiology at http://www.epidem.com/). Information on the STROBE Initiative is available at http://www.strobe-statement.org.
